# Radon in Schools: A Brief Review of State Laws and Regulations in the United States

**DOI:** 10.3390/ijerph15102149

**Published:** 2018-09-30

**Authors:** Kelsey Gordon, Paul D. Terry, Xingxing Liu, Tiffany Harris, Don Vowell, Bud Yard, Jiangang Chen

**Affiliations:** 1 Department of Public Health, 390 HPER Building, 1914 Andy Holt Avenue, University of Tennessee, Knoxville, TN 37996, USA; kgordo10@utk.edu (K.G.); xliu92@vols.utk.edu (X.L.); tloveda4@vols.utk.edu (T.H.); 2 Department of Medicine, The University of Tennessee Medical Center, Knoxville, TN 37920, USA; 3 The Vowell Law Firm, 6718 Albunda Drive, Knoxville, TN 37919, USA; don@vowell-law.com; 4 Tennessee Department of Environment and Conservation, 761 Emory Valley Rd, Oak Ridge, TN 37830, USA; Bud.Yard@tn.gov

**Keywords:** radon exposure, environmental health of schools, state policy

## Abstract

Exposure to Radon, a colorless, naturally occurring radioactive gas, is one of leading causes of lung cancer, and may pose a significant long-term risk for school age children. We examined the regulations and statutes in each US state related to radon in schools to delineate key features of policies and discrepancies among states that may have public health implications. Search terms such as “radon”, “school”, “mitigation”, “certification”, “licensing”, and “radon resistant new construction” were used to scan current statutes from each state legislature’s website and regulations from official state government websites for relevant regulatory and statutory requirements concerning radon in schools. State regulations related to the testing, mitigation, and public dissemination of radon levels in schools are inconsistent and the lack of nationwide indoor radon policy for schools may result in unacceptably high radon exposure levels in some US schools. We highlight the features and discrepancies of state laws and regulations concerning radon in schools, and offer several constructive means to reduce risks associated with radon exposure in school children.

## 1. Introduction

Radon is a naturally occurring colorless and odorless gas. It was first discovered in the form of ^222^Rn in 1899, with two other isotopes of Radon (^220^Rn and ^219^Rn) discovered subsequently [1]. In general, ^219^Rn and ^220^Rn are not significant public health concerns in modern, well-maintained architectural structures [2] due to the short half-life (3.96 and 55.6 seconds respectively). ^222^Rn, in contrast, has a half-life of 3.8 days, which allows it to travel some distance. ^222^Rn can seep into building through cracks in floors, construction joints, and/or around service pipes. Henceforward, ^222^Rn is referred to simply as ’’radon’’. There is no safe level of radon in the living environment.

Health effects of radon, most notably lung cancer, have been investigated for several decades. Initially, investigations focused on underground miners exposed to high concentrations of radon in their occupational environment [3]. However, the results of several surveys of radon concentrations in homes and other buildings in the early 1980s suggested that radon also may be an important cause of lung cancer in the general population [4]. Krewski and colleagues evaluated the risk associated with prolonged residential radon exposure and risk of lung cancer using data collected from seven large scale case-control studies (4081 cases and 5281 controls) conducted in North America [5]. The estimated pooled odds ratio (OR) of lung cancer increased with radon concentration monotonically [5]. Analysis from thirteen case-control studies conducted in nine European countries between 1982–1995 also showed appreciable hazards from residential radon exposure, responsible for an estimated 2% of all deaths from cancer in Europe [6]. The risk of lung cancer increased by 8.4% per 100 Bq/m^3^ (~2.7 pCi/L) increase in measured radon [6]. These residential studies provide evidence of a positive association between residential radon exposure and lung cancer risk [7]. Currently, the World Health Organization (WHO) and the United States Environmental Protection Agency (USEPA) both classify radon as a leading causes of lung cancer, second only to smoking.

Radon exposure at schools may have a considerable public health impact. It was estimated that approximately 14% of the 300,000 annual lung cancer cases in the United States are attributable to radon [8]. The risk of lung cancer in children resulting from exposure to radon may be up to three- fold higher than that of adults exposed to the same amount of radon [9] due to the morphometric differences between the lungs of children and the lungs of adults [10], as well as higher respiration rates of children compared with adults. Children also spend more time indoors, and are generally more sensitive to environmental hazards exposure [11]. It was estimated that U.S. children, on average, spend 6.64 h per day for 180 days per year in school buildings [12]. This does not include the substantial additional hours that children might spend in school buildings in after-school programs. Schools are also workplaces for teachers and administrators and service staff, who might spend even more time than children in school buildings. In the late 1980s, short-term radon measurements were conducted in 3000 rooms in 130 U.S. schools in 16 states [13]. Over half of the schools had at least one room with radon levels above 4 pCi/L, and all 16 tested states had some classrooms with radon above 4 pCi/L, with the highest classroom showing 136.2 pCi/L. Therefore, high levels of radon in school buildings may pose significant health risks to those who spend many months, or years, at those schools [14].

Given the lack of U.S. Federal regulations concerning radon levels in schools, we reviewed specific state statutes on radon that could influence exposure to school children, including bills that passed in the 2017 U.S. legislative sessions. We further discuss Federal and state regulations as they relate to radon reduction and control, radon testing, dissemination of radon levels to the public, radon mitigation, and various challenges and policy recommendations.

## 2. Radon Reduction and Control: Federal Laws and Efforts

Radon exposure has not been linked with major diseases in childhood, but the risk of greatest concern is that of consequent lung cancer in adulthood. The USEPA’s risk estimates for radon are based on cohort studies of male workers in mines [7,15]; however, the derived risk estimates were erroneously extrapolated to women, children, and non-working men. Cao and associates recently re-analyzed cohort studies of male workers in mines, and the results show that the USEPA’s original estimates of fatal risks attributable to radon may be overestimated by 9–26% after accounting for confounding by occupational exposure to diesel. Although methods for estimating risks have changed over the years, the observed risks attributable to radon are still considered to be unacceptable [7], and radon exposure maintains its high public health importance as the second leading cause of lung cancer deaths in the U.S. Whereas European Union (EU) regulations regarding radon are discussed briefly in a separate section below, we note here that the influence of EU directives on radon exposure in EU member countries may have relevance to the role U.S. Federal agencies, such as the USEPA, may play in reducing exposure in individual U.S. states [16].

The Superfund Amendments and Reauthorization Act (SARA) of 1986 requires USEPA to (1) conduct nationwide assessments of radon gas across the United States where people live, work, and go to school; (2) assess the levels of radon gas present in such structures and the impact to human health; and (3) determine methods of reducing or eliminating human exposure to radon gas and provide guidance and public information materials based on the results of those assessments (SARA, Title I, Section 118(k) (1)). SARA further requires the USEPA to (1) establish a research program to facilitate the understanding of health problems associated with exposure to air pollutants including radon gas in the indoor environment; (2) coordinate Federal, state, local, and private research and development efforts to improve indoor air quality; and (3) assess appropriate Federal Government actions to mitigate the environmental and health risks associated with indoor air quality problems. Following the SARA of 1986, the Indoor Radon Abatement Act of 1988 granted USEPA authority to conduct radon studies and compile a list of high risk areas, including schools, with elevated levels of radon.

Over twenty years after the passing of SARA and the Indoor Radon Abatement Act, public understanding of the gravity of the radon risk remains limited [17,18,19]. Further, the perceived high costs of radon mitigation, and the relative shortage of certified radon tests and mitigation professionals, impedes the reduction of indoor radon exposure [20]. Consequently, under the leadership of USEPA, the Federal Radon Action Plan (FRAP), a collaborative inter-agency program, was initiated in 2011 to unify federal programs, disseminate radon exposure health risk information to the public, provide professional radon testing and mitigating services, and promote radon risk reduction through financial incentives and support [21]. Since the initiation of FRAP, at least 1.6 million homes, schools, and childcare facilities benefited, and in 12.5% of those units, testing and mitigating were conducted when necessary as of 2014. However, the data are not available to the public on exactly how many schools were benefited from FRAP. Because FRAP was phased out in 2015, the National Radon Action Plan (NRAP), led by the American Lung Association, has assumed several of FRAP’s functions for radon risk reduction, with the goal of mitigating 5 million ’’high radon’’ homes and saving 3200 lives from lung cancer annually by 2020 [21]. NRAP however, has no explicit mandate to mitigate ’’high radon’’ schools. Even the Indoor Radon Abatement Act has not stipulated an enforceable indoor radon limit for schools but only recommends a national goal that ’’the air within buildings in the United States should be as free of radon as the ambient air outside of buildings’’. A nationwide survey of radon levels in schools estimated that nearly one in five schools in the US has at least one schoolroom with short-term radon levels above the action level of 4 pCi/L, the level at which USEPA recommends that schools take mitigation actions [22]. In New York state, for instance, approximately 90% of the 4290 upstate schools are located in area designated by USEPA as Zone 1 (4 pCi/L) [13]. Since 1992, the New York Department of Health has conducted radon concentration measurement in over 12,300 classrooms in 186 schools using short-term detectors. Eighteen schools had >4 pCi/L of radon in over half of the rooms. About 1150 school rooms, or 9.3% of those measured, contained from 4 pCi/L to 20 pCi/L of radon, and a smaller number of school rooms in 19 schools contained up to 80.2 pCi/L of radon [13]. USEPA estimates that more than 70,000 schoolrooms in use today have high short-term radon levels [22].

## 3. Radon Reduction and Control in Schools: State Laws and Regulations

### 3.1. State Radon Testing in School Requirements

Currently, only thirteen states’ statutes explicitly address radon issues in schools, and radon testing in schools is only mandatory in nine states (Table 1). For example, radon testing in schools is not mandatory in California, a state that has over 10,000 public schools (elementary and secondary) with over 6.3 million students [23]. Even within these nine states, state regulations on radon testing in schools vary considerably in stringency. Connecticut statutes, for example, require radon testing in public schools only, whereas in New York, radon testing is only required for public schools located in Radon Zone 1. In contrast, radon testing is mandatory for all schools (Public and Private) in Rhode Island. The timing and frequency of radon re-testing also differ among the states (Table 1).

### 3.2. State Test Results Dissemination Requirements

State statutes and code regulations also differ in the dissemination of radon testing results to the public. For example, a Colorado statute requires all schools to test for radon and to maintain records of the test results for disclosure on request. In other words, schools in Colorado are not required to share test results with the state agency that regulates radon tests in schools; nor are they required to report the results to teachers or parents [24], unless such request were made.

A nationwide policy is therefore essential to mandate radon test reports to be submitted to a designated state regulatory agency, to be disseminated to parents, other interested parties and the district school board members, as well as to make the results available on the school’s or district’s website. In Connecticut, for example, the school radon testing guide recommends that the school administration conducts an informational meeting to provide an overview of scheduled radon testing and address any questions/concerns to individuals responsible for radon testing and representatives of parent and teacher organizations. In addition, in Connecticut, it is recommended that two weeks prior to a scheduled radon testing, a formal letter of radon testing notification is required to be sent to parents of students and school employees, along with radon educational materials. Proper notification, in a transparent and timely way, will encourage civil society stakeholders to voice their concerns and participate in monitoring policy and program implementation.

### 3.3. State Radon Mitigation Requirements

In the United States, a radon level below 2 pCi/L is accepted as normal (Radon Zone 3). Indoor Radon level between 2 and 4 pCi/L is designated as Radon Zone 2 and the USEPA suggests mitigation to be performed. Indoor radon level of above 4 pCi/L (Action Level) is categorized as Radon Zone 1 where mitigation is deemed necessary because an increased risk for lung cancer has been observed at that exposure level in smokers and non-smokers [25]. However, radon mitigation requirements in most states varies substantially and often are not enforced (Table 1). For example, the statute of state of Colorado doesn’t require schools to mitigate, so it’s up to the school district and its constituents to address mitigation issues [24].

## 4. Radon Regulations in Europe

Although not the main focus of our review, radon in schools should be a concern in any geographic region where levels are above those deemed to be safe. The EU recently enacted a directive establishing 300 Bq/m^3^ (~8.1 pCi/L) as a concentration threshold for both dwellings and workplaces including schools in all European Union countries [16,26]. In Ireland, for example, remedial work is required for any occupied classroom or office where the radon levels are greater than 200 Bq/m^3^ (~5.4 pCi/L) [27,28,29]. In a pilot study conducted in Spain, 46% of the workplaces measured in Galicia had radon concentrations higher than 300 Bq/m^3^, followed by 10.6% in Madrid. Nineteen percent of all workers were exposed to more than 300 Bq/m^3^, with 6.3% exposed to radon concentrations higher than 500 Bq/m^3^ [26]. Nationwide radon maps based on indoor measurements have been produced in countries such as the United Kingdom, Ireland, and Italy [4,30], which may facilitate and optimize the search for schools with high radon concentrations and to identify areas for special preventive actions during new construction [4,13,31]. In Italy, the Legislative Decree 241/2000 set 500 Bq/m^3^ as the highest acceptable radon concentration in workplaces including schools [30]. With the 2013/59/Euratom Directive, the obligation is introduced for member countries to periodically prepare and update a Radon National Plan in order to implement the set of actions necessary to reduce the health risk associated with radon [30]. However, it remains to be seen whether certain barriers may prevent some EU member countries from fully complying with the Directive. In time, this Directive may serve as a model for standardized national U.S. regulations as they apply to radon testing and mitigation.

## 5. Challenges and Policy Recommendations

One of the biggest challenges related to radon exposure is the cost of testing and mitigation. If conducted by licensed professionals, which is required in several states, testing could cost $20–$50 per room, and the sub-slab depressurization, the most common type of radon mitigation system, could cost between $2000 to $5000, depending on characteristics of the building and the underlying soil [32]. This could pose a substantial burden for some schools, school districts, and/or state agencies that already are struggling to meet other mandatory regulations and maintain their financial sustainability. In California, for example, there is no state funding specifically set aside to help school districts pay for testing [33]. One of the approaches to reduce radon testing costs to the school could be allowing school employees to take a one-day radon test training and be exempt from certification requirements; alternately, after training, school employees in the same district could jointly form a radon testing team, therefore schools could conduct initial testing on their own. When initial tests results raise an alarm, the certified professionals would then be called in for a follow up testing, as has been done in New Jersey and Illinois (Table 1).

In addition, each state should establish a designated Fund as a non-lapsing supplement to support radon testing, educational, and mitigation. For example, in Indiana (Radon Gas Trust Fund) and Maine (Radon Relief Fund), statutes allow the States to collect funds received from registrations of radon testing and mitigation companies/professionals, as well as any other miscellaneous sources of income to be used for a radon related program.

## 6. Conclusions

The danger to children attending schools with high levels of radon is particularly noteworthy because such children would be exposed repeatedly over many years. They would also be exposed during a biologically susceptible time of life and would subsequently have many years during which cancer may develop and have a clinical impact (a long ’’latency’’ periods). To date, however, the issue of radon in schools has received little attention by researchers and the media. There is currently no enforceable Federal limit for radon levels in schools, which has led to disparate state regulations related to the testing, mitigation, and public dissemination of radon levels, as well as the appropriation of state funds when mitigation is needed.

From a public health perspective, radon reduction is a long-term objective. Widespread awareness among the general population about the risk associated with radon is essential [34]. It is also critical to improve the data collection system in each state to make data more accessible to advocates for radon testing, to societal stakeholders, and to the general public [24,35]. The improvements in data collection and availability will be valuable for future campaigns and for determining where collaborative efforts are most needed [35]. Measuring and mitigating radon at schools should be based on ALARA (As Low As Reasonably Achievable), the precautionary principle to minimize radiation doses and releases of radioactive materials with using practical, cost-effective measures [4,36,37]. Therefore, we recommend that a federally mandated maximum radon limit be established for schools and other buildings with public access, as should guidelines regarding the timeframe for initial testing and the frequency of re-testing for radon. States may then work with the USEPA to develop a state-specific radon testing plans based on the risk related to the specific geographic locations of the schools. The USEPA may also offer standardized online radon testing training courses and materials for school employees to reduce state costs and improve the consistency, accuracy, and reproducibility of the test results.

## Figures and Tables

**Table 1:** Radon testing in schools: State policies.

State	% of Counties Designated as EPA Zone 1 or Zone 2 ^a^	Radon Test	Time Requirement for Initial Testing	Frequency of Re-Testing	Type of School Covered		Who May Conduct the Testing		New School RRNC Requirement	Mitigation Requirement for Radon Level ≤ 4 pCi/L
					Public	Private	SE	CP		
Colorado	100	Required	Within 19 months of occupancy	ND	Yes	Yes	ND	ND	No	No
Connecticut	87.5	Required	ND	Every 5 years	Yes	No	No	Yes	Yes	Yes
Florida	13.4	Required ^b^	Within 12 months of occupancy	Twice, 5 years apart	Yes	Yes	No	Yes	No	No
Illinois	100	Recommend	ND	Every 5 years	Yes	No	Yes	Yes	No	No
Iowa	100	Encourage	ND	ND	Yes	Yes	No	Yes	No	No
Minnesota	100	Voluntary	ND	ND	Yes	No	Yes	Yes	No	Yes ^c^
New Jersey	85.7	Required	ND	Every 5 years	Yes	No	Yes	Yes	Yes	No
New York	75.8	Required ^d^	ND	ND	Yes	No	No	Yes	No	Yes
Oregon	58.3	Required	ND ^e^	Every 10 years	Yes	No	Yes	Yes	Yes	No
Rhode Island	80	Required	ND	Every three years ^f^	Yes	Yes	No	Yes	No	Yes
Tennessee	77.8	Encourage	ND	ND	Yes	No	ND	ND	No	No
Virginia	71.6	Required	With 18 months of occupancy	ND	Yes	No	Yes	Yes	No	Yes
West Virginia	87.5	Required	With 12 month of occupancy	Every 5 years	Yes	No	No	Yes	Yes	Yes

ND: Not Defined; SE: School Employees; CF: Certified Professionals. ^a^: Based on USEPA Radon Map https://www.epa.gov/radon/find-information-about-local-radon-zones-and-state-contact-information#radonmap. ^b^: All schools in continuous use since before 1 July 1994 must test 100% first floor or below grade rooms, 20% second floor rooms, and 10% third floor rooms; For school opened between 1 July 1994 and 30 June 1999 must test 100% first floor; For school opened or structurally changed since 1 July 1999, 20% of ground contact rooms must be tested. ^c^: School districts that receive health and safety revenue to conduct radon testing must take re-test after corrective measures that reduce radon levels. ^d^: School should review geological potential for radon and to decide if test is necessary. Radon testing is only required for public schools located in Radon Zone 1. ^e^: Statewide radon testing in school must be completed on or before 1 January 2021. ^f^: If initial testing is ≤4 pCi/L, 10% of the areas must be re-tested 3 years after; A different 10% of the building must be tested every 3 years thereafter.

## References

[1] Thornton B.F., Burdette S.C. (2013). Recalling Radon’s Recognition. Nat. Chem..

[2] Meisenberg O., Mishra R., Joshi M., Gierl S., Rout R., Guo L., Agarwal T., Kanse S., Irlinger J., Sapra B.K. (2017). Radon and Thoron Inhalation Doses in Dwellings with Earthen Architecture: Comparison of Measurement Methods. Sci. Total Environ..

[3] Loring D.M. A study of radon regulation and pathology as it relates to underground hard rock mining. Proceedings of the 12th U.S./North American Mine Ventilation Symposium, Society for Mining, Metallurgy and Exploration Inc..

[4] World Health Organization (WHO) (2009). WHO Handbook on Indoor Radon: A Public Health Perspective.

[5] Krewski D., Lubin J.H., Zielinski J.M., Alavanja M., Catalan V.S., Field R.W., Klotz J.B., Létourneau E.G., Lynch C.F., Lyon J.L. (2006). A combined analysis of North American case-control studies of residential radon and lung cancer. J. Toxicol. Env. Health Part A.

[6] Darby S., Hill D., Auvinen A., Barros-Dios J.M., Baysson H., Bochicchio F., Deo H., Falk R., Forastiere F., Hakama M. (2005). Radon in homes and risk of lung cancer: collaborative analysis of individual data from 13 European case-control studies. Br. Med. J..

[7] Cao X., MacNaughton P., Laurent J.C., Allen J.G. (2017). Radon-induced lung cancer deaths may be overestimated due to failure to account for confounding by exposure to diesel engine exhaust in BEIR VI miner studies. PLoS ONE.

[8] United States Environmental Protection Agency (USEPA) News Releases by State. Radon Action Could Save Many Lives 01/07/2009. https://archive.epa.gov/epapages/newsroom_archive/newsreleases/86ef1e837f6b5f858525753700654f9e.html.

[9] National Council on Radiation Protection and Measurements (NCRP) (1984). Evaluation of Occupational and Environmental Exposures to Radon and radon daughters in the United States. https://ncrponline.org/shop/reports/report-no-078-evaluation-of-occupational-and-environmental-exposures-to-radon-and-radon-daughters-in-the-united-states-1984/.

[10] Samet J.M. (1989). Radon and lung cancer. J. Natl. Cancer Inst..

[11] Grigg J. (2004). Environmental toxins; their impact on children’s health. Arch. Dis. Child..

[12] National Center for Education Statistics (2007–2008): School and Staffing Survey. https://nces.ed.gov/surveys/sass/tables/sass0708_035_s1s.asp.

[13] Kitto M. (2014). Radon testing in schools in New York State: A 20-year summary. J. Environ. Radioact..

[14] Foster S., Jones S.E. (2016). Association of School District Policies for Radon Testing and Radon-Resistant New Construction Practices with Indoor Radon Zones. Int. J. Environ. Res. Public Health.

[15] National Research Council (NRC) (1999). Health Effects of Exposure to Radon: BEIR VI.

[16] (2014). European Council Directive 2013/59/Euratom on Basic Safety Standards for Protection against the Dangers Arising from Exposure to Ionising Radiation and Repealing Directives 89/618/Euratom, 90/641/Euratom, 96/29/Euratom, 97/43/Euratom and 2003/122/Euratom. Off. J. Eur. Union.

[17] Duckworth L.T., Frank-Stromborg M., Oleckno W.A., Duffy P., Burns K. (2002). Relationship of perception of radon as a health risk and willingness to engage in radon testing and mitigation. Oncol. Nurs. Forum.

[18] Hill W.G., Butterfield P., Larsson L.S. (2006). Rural parents’ perceptions of risks associated with their children’s exposure to radon. Public Health Nurs..

[19] Vogeltanz-Holm N., Schwartz G.G. (2018). Radon and lung cancer: What does the public really know?. J. Environ. Radioact..

[20] United States Environmental Protection Agency (USEPA) Protecting People and Families from Radon: A Federal Action Plan for Saving Lives. https://www.epa.gov/sites/production/files/2014-08/documents/Federal_Radon_Action_Plan.pdf.

[21] United States Environmental Protection Agency (USEPA) The National Radon Action Plan: A Strategy for Saving Lives. https://www.epa.gov/radon/national-radon-action-plan-strategy-saving-lives.

[22] United States Environmental Protection Agency (USEPA) Radon Measurement in Schools Revised Edition. https://www.epa.gov/iaq-schools/radon-measurement-schools-revised-edition.

[23] National Center for Education Statistics National Center for Education Statistics (2014–2015): State Education Data Profile. https://nces.ed.gov/programs/stateprofiles/sresult.asp?mode=short&s1=06.

[24] Colorado State Department of Public Health and Environment Colorado State Department of Public Health and Environment: Radon in Schools. https://www.colorado.gov/pacific/cdphe/understanding-radon.

[25] Lantz P.M., Mendez D., Philbert M.A. (2013). Radon, Smoking, and Lung Cancer: The Need to Refocus Radon Control Policy. Am. J. Public Health..

[26] Ruano-Ravina A., Narocki C., López-Jacob M.J., Oliver A.G., Calle Tierno M.C., Peón-González J., Barros-Dios J.M. (2018). Indoor radon in Spanish workplaces. A pilot study before the introduction of the European Directive 2013/59/Euratom. Gac. Sanit..

[27] McGarry A.T. (1996). Practical implications of radon regulation in Ireland. Annales l’Association Belge Radioprotection.

[28] Ireland Environmental Protection Agency National Radon Control Strategy: Schools. http://www.epa.ie/radon/schools/.

[29] Synnott H., Colgan P.A., Hanley O., Fenton D. (2007). The effectiveness of radon remediation in Irish schools. Health Phys..

[30] Azara A., Dettori M., Castiglia P., Piana A., Durando P., Parodi V., Salis G., Saderi L., Sotgiu G. (2018). Indoor Radon Exposure in Italian Schools. Int. J. Environ. Res. Public Health.

[31] Synnott H., Hanley O., Fenton D., Colgan P.A. (2006). Radon in Irish schools: The results of a national survey. J. Radiol. Prot..

[32] State of New Jersey Department of Education Radon Testing in Schools. http://www.state.nj.us/education/facilities/memos/radon.pdf.

[33] California Department of Public Health California Department of Public Health: Radon in Schools. https://www.cdph.ca.gov/Programs/CEH/DRSEM/Pages/EMB/Radon/Radon-in-Schools.aspx.

[34] Lecomte J.F., Solomon S., Takala J., Jung T., Strand P., Murith C., Kiselev S., Zhuo W., Shannoun F., Janssens A. (2014). International Commission on Radiological Protection (ICRP): Radiological Protection against Radon Exposure. Annals ICRP.

[35] Bain A.A., Abbott A.L., Miller L.L. (2016). Successes and Challenges in Implementation of Radon Control Activities in Iowa, 2010–2015. Prev. Chron. Dis..

[36] García-Talavera M., Martínez M., Matarranz J.L., Ramos L. (2008). Setting radon-specific release criteria and demonstrating compliance for land affected by NORM. Appl. Radiat. Isot..

[37] Branion-Calles M.C., Nelson T.A., Henderson S.B. (2015). Evaluation of different radon guideline values based on characterization of ecological risk and visualization of lung cancer mortality trends in British Columbia, Canada. BMC Public Health.

